# Inhibitory Effects of *N*-[2-(4-acetyl-1-piperazinyl) phenyl]-2-(2-chlorophenoxy) acetamide on Osteoclast Differentiation In Vitro via the Downregulation of TRAF6

**DOI:** 10.3390/ijms20205196

**Published:** 2019-10-20

**Authors:** Zhihao Chen, Eunjin Cho, Jinkyung Lee, Sunwoo Lee, Tae-Hoon Lee

**Affiliations:** 1 Department of Molecular Medicine (BK21plus), Chonnam National University Graduate School, Gwangju 61186, Korea; chinaczhihao@gmail.com; 2 Department of Oral Biochemistry, Dental Science Research Institute, School of Dentistry, Chonnam National University, Gwangju 61186, Korea; ag8414@gmail.com (E.C.); wlsrud1945@naver.com (J.L.); 3 Department of Chemistry, Chonnam National University, Gwangju 61186, Korea; sunwoo@chonnam.ac.kr

**Keywords:** piperazine, osteoclastogenesis, TRAF6, bone resorption

## Abstract

Osteoclasts are poly-nuclear cells that resorb mineral components from old or damaged bone tissue. Primary mononuclear cells are activated by receptor activator of nuclear factor kappa-Β ligand (RANKL) and differentiate into large multinucleated cells. Dysregulation of osteoclast differentiation can lead to pathological bone loss and destruction. Many studies have focused on the development of new molecules to regulate RANKL-mediated signaling. In this study, *N*-[2-(4-acetyl-1-piperazinyl)phenyl]-2-(2-chlorophenoxy) acetamide (PPOA-*N*-Ac-2-Cl) led to a significant decrease in the formation of multinucleated tartrate-resistant acid phosphatase (TRAP)-positive cells in a dose-dependent manner, without inducing significant cytotoxicity. PPOA-*N*-Ac-2-Cl affected the expression of osteoclast-specific marker genes, such as *TRAF6*, *c-fos, DC-STAMP*, *NFATc1*, *MMP9*, *CtsK*, and *TRAP (Acp5)*, during RANKL-mediated osteoclastogenesis. Moreover, PPOA-*N*-Ac-2-Cl significantly attenuated the protein levels of *CtsK*, a critical protease involved in bone resorption. Accordingly, bone resorption activity and F-actin ring formation decreased in the presence of PPOA-*N*-Ac-2-Cl. In conclusion, this study shows that PPOA-*N*-Ac-2-Cl acts as an inhibitor of osteoclast differentiation and may serve as a potential candidate agent for the treatment of osteoclast-related bone diseases by virtue of attenuating bone resorption.

## 1. Introduction

Bone homeostasis is finely regulated by osteoblasts that form bone and osteoclasts that absorb bone. An imbalance in this homeostasis because of various abnormal factors can cause different bone diseases. In particular, an extreme enhancement in osteoclast activation can lead to bone loss-mediated osteoporosis and osteolytic diseases [1]. Thus, targeting osteoclast differentiation or the functions of osteoclast can be effective strategies to treat bone diseases with abnormally enhanced activation of osteoclasts [2,3]. Evidence has shown that NF-κB, c-Src, and mitogen-activated protein kinases (MAPKs)-mediated signaling pathways are critical for osteoclast differentiation and require intermediate cytokines, such as receptor activator of nuclear factor kappa-B ligand (RANKL), interleukin-1 (IL-1), and tumor necrosis factor alpha (TNF-α) [4]. Briefly, interaction between RANKL and receptor activator of nuclear factor kappa-B (RANK) leads to recruitment of TRAF6, which can activate multiple critical signaling pathways, including NF-κB and Ca^2+^ signaling, thereby activating the expression of genes involved in the differentiation and function of osteoclasts. In addition, suppression of extracellular signal-regulated kinase (ERK), p38, and c-Jun N-terminal kinase (JNK) induces inhibition of RANKL-activated osteoclastogenesis [5]. Phosphoinositide 3-kinase (PI3k)/Akt also plays an important role in the differentiation and function of osteoclast [6,7]. Activation of these signaling pathways induces nuclear factor of activated T-cells cytoplasmic1 (NFATc1), which eventually triggers the expression of osteoclast-specific genes, such as tartrate-resistant acid phosphatase (*TRAP*), dendritic cell-specific transmembrane protein (*DC-STAMP*), matrix metalloproteinase- 9 (*MMP-9*), and cathepsin K (*CtsK*) [8].

In recent years, molecular-level studies on the relationship between osteoclastic bone resorption and osteoblastic bone formation have led to the discovery of various therapeutic compounds. Bisphosphonate, the most commonly prescribed anti-resorptive drug, has been reported to inhibit bone resorption via targeting osteoclast apoptosis and osteoclast function [9,10]. However, treatment with bisphosphonate can lead to severe side effects, such as osteonecrosis in the jaw and hypocalcemia [11,12]. Therefore, alternative therapies are necessary to avoid such side effects. The derivatives of *N*-(piperazine-1-yl)phenyl-2-phenoxyacetamide (PPOA), shown in Figure S1, have been reported to show biological and pharmacological functions. Acetamide A and B, which carry acyl groups at the piperazine moiety, have been reported as both sirtuin-modulating compounds and antitumor agents [13,14]. Acetamide C, containing a methyl group at the piperazine moiety, has been reported as an antitumor agent [15]. In addition, acetamide D, containing a methylsulfonyl group, has been employed as a candidate tyrosine-sulfate mimetic [16]. However, little is known about the effect of PPOAs on osteoclastogenesis and osteolytic diseases.

In this study, we investigated whether PPOA derivatives could attenuate RANKL-induced osteoclast differentiation in vitro and examined the molecular mechanisms underlying the inhibitory effects of PPOA-*N*-Ac-2-Cl during osteoclastogenesis. PPOA derivatives, especially PPOA-*N*-Ac-2-Cl, significantly inhibited osteoclast differentiation. Further experiments indicated that PPOA-*N*-Ac-2-Cl repressed RANKL-induced osteoclast formation at the early stages of osteoclastogenesis, without inducing significant cytotoxicity. PPOA-*N*-Ac-2-Cl disrupted F-actin ring formation at a concentration of 6 µM in vitro. In addition, PPOA-*N*-Ac-2-Cl attenuated bone resorption during osteoclastogenesis in a dose-dependent manner; further, it suppressed the expression levels of osteoclast-related marker genes, such as *TRAF6*, *c-fos*, *DC-STAMP*, *ATP6v0d2*, *NFATc1*, *MMP9*, *CtsK*, and *Acp5*. Collectively, these results suggest that PPOA-*N*-Ac-2-Cl inhibits osteoclast formation and bone resorption, indicating its potential as a candidate agent for the treatment of bone resorption-related diseases.

## 2. Results

### 2.1. PPOA-N-Ac-2-Cl Inhibits RANKL-Induced Osteoclast Differentiation

**Figure 1:**
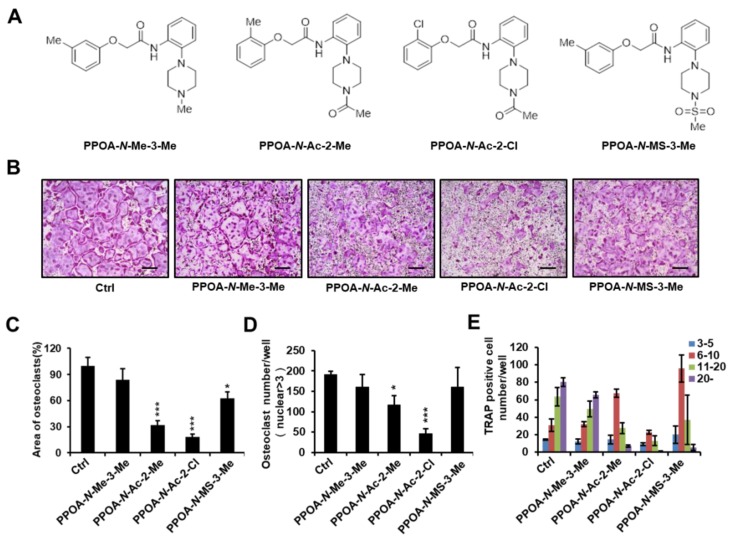
(**A**) Structures of PPOA-*N*-Me-3-Me, PPOA-*N*-Ac-2-Me, PPOA-*N*-Ac-2-Cl, and PPOA-*N*-MS-3-Me. (**B**) Bone marrow monocytes (BMMs) were differentiated into osteoclasts by treatment with macrophage colony-stimulating factor (M-CSF) (30 ng/mL) and receptor activator of nuclear factor kappa-Β ligand (RANKL) (50 ng/mL) in the presence of PPOA derivatives (5 μM each). (**C**–**E**) Tartrate-resistant acid phosphatase (TRAP)-positive cells possessing more than three nuclei in panel (**B**) were calculated using Image J software. Scale bar = 200 μm. The spread areas (**C**) and number (**D**,**E**) of TRAP-positive cells were calculated using Image J. * *p* < 0.05, and *** *p* < 0.001 indicate the statistically significant difference compared to vehicle-treated control.

To investigate whether PPOA compounds affect osteoclastogenesis, we selected four PPOA derivatives (Figure 1A) from our in-house-synthesized compounds, based on their structural properties. An initial screen was performed with these compounds. Mouse bone marrow monocytes (BMMs) were incubated with or without 5 μM PPOA-*N*-Me-3-Me, PPOA-*N*-Ac-2-Me, PPOA-*N*-Ac-2-Cl, or PPOA-*N*-MS-3-Me in the presence of macrophage colony-stimulating factor (M-CSF) (30 ng/mL) and RANKL (50 ng/mL) for 3 days to induce osteoclast differentiation. We observed that, among all the tested compounds, PPOA-*N*-Ac-2-Cl elicited the most significant decrease in the total area of osteoclasts, upon TRAP staining (Figure 1B,C). We calculated the TRAP-positive multinucleated cells (cells with nuclei > 3) based on the nuclei number (Figure 1D,E). The total number of osteoclasts and multinucleated osteoclasts possessing more than six nuclei were significantly reduced after treatment with PPOA-*N*-Ac-2-Cl. Therefore, we selected PPOA-*N*-Ac-2-Cl for further experiments to investigate its effect on osteoclastogenesis.

**Figure 2:**
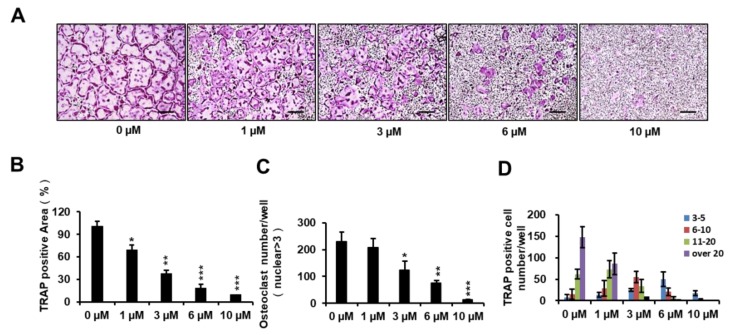
PPOA-*N*-Ac-2-Cl inhibits RANKL-induced osteoclastogenesis in a dose-dependent manner. (**A**) Inhibitory effect of PPOA-*N*-Ac-2-Cl on osteoclastogenesis, as observed by TRAP staining. BMMs were cultured with various concentrations of PPOA-*N*-Ac-2-Cl (0, 1, 3, 6, and 10 μM) for 4 days in the induction medium, containing M-CSF (30 ng/mL) and RANKL (50 ng/mL); subsequently, TRAP staining was performed to visualize osteoclast formation. Scale bar = 200 μm. The area (**B**), number (**C**) of TRAP-positive multinuclear cells and the TRAP positive cell number based on the number of nuclei (**D**) were calculated. * *p* < 0.05, ** *p* < 0.01, and *** *p* < 0.001 versus vehicle-treated control (0 μM).

To determine the inhibitory effect of PPOA-*N*-Ac-2-Cl on osteoclastogenesis, BMMs were treated with PPOA-*N*-Ac-2-Cl at 0, 1, 3, 6, and 10 μM. As shown in Figure 2A, an evident reduction in the percentage of TRAP-positive cells was observed when osteoclasts were treated with PPOA-*N*-Ac-2-Cl, and this inhibition occurred in a dose-dependent manner. In addition, the area and the number of TRAP-positive osteoclasts (cells with nuclei > 3) reduced significantly upon treatment with PPOA-*N*-Ac-2-Cl at the indicated concentrations (Figure 2B,C). Moreover, the number of nuclei per osteoclast reduced dramatically following treatment with increasing concentrations of PPOA-*N*-Ac-2-Cl (Figure 2D). To exclude the possibility that the suppressive effect of PPOA-*N*-Ac-2-Cl on osteoclastogenesis was because of cytotoxicity, cell viability was analyzed by the 3-(4,5-dimethylthiazol-2-yl)-2,5-diphenyltetrazolium bromide (MTT) colorimetric assay. The results showed that PPOA-*N*-Ac-2-Cl exhibited no cytotoxic effect after 72 h of treatment at concentrations of 1, 3, 6, and 10 μM (Figure S2). Hence, the results suggested that PPOA-*N*-Ac-2-Cl inhibited osteoclastogenesis without inducing any cytotoxicity. 

### 2.2. PPOA-N-Ac-2-Cl Inhibits Osteoclast Differentiation Only at the Early Stage 

**Figure 3:**
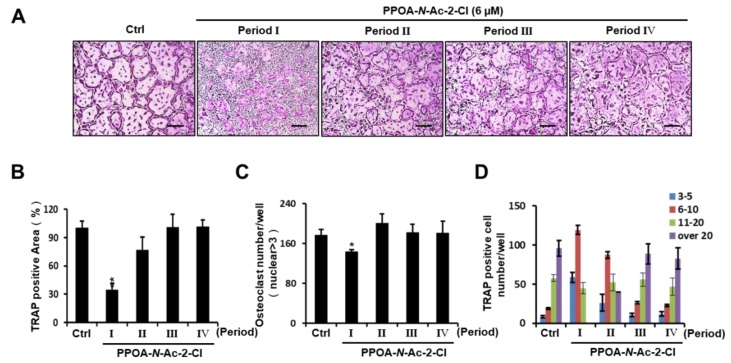
PPOA-*N*-Ac-2-Cl inhibits osteoclastogenesis at early stages of osteoclast differentiation. (**A**) BMMs were seeded for five groups (Ctrl, and Periods I–IV) and cultured in the induction medium, containing M-CSF (30 ng/mL) and RANKL (50 ng/mL) for four days. The BMMs of Periods I–IV were treated with 6 μM PPOA-*N*-Ac-2-Cl for 24 h on different days, day 1, 2, 3, or 4, respectively. After four days, the cells in all groups were fixed and TRAP staining was performed to determine the presence of osteoclasts. Scale bar = 200 μm. (**B**–**D**) Graphs indicating the calculation of panel (**A**). The area (**B**) and total number (**C**) of TRAP-positive cells, and TRAP positive cell number based on the number of nuclei (**D**) were calculated using Image J software. * *p* < 0.05 versus the control group.

To assess whether PPOA-*N*-Ac-2-Cl inhibits osteoclast differentiation in a temporal manner, we examined the appearance of TRAP-positive cells during RANKL-induced osteoclastogenesis upon treatment with PPOA-*N*-Ac-2-Cl for the indicated periods (Periods I–IV). As shown in Figure 3A, an obvious reduction in the number of multinucleated giant cells was observed only on the first day of PPOA-*N*-Ac-2-Cl treatment (Period I). When we analyzed TRAP-positive osteoclasts, compared to the control, the area and number of osteoclasts were found to decrease significantly during Period I, but were not altered significantly during other Periods (Figure 3B,C). Moreover, osteoclasts containing more than 20 nuclei were not detected during Period I (Figure 3D). Collectively, these results indicate that PPOA-*N*-Ac-2-Cl suppressed RANKL-induced osteoclast formation at the early stages of differentiation.

### 2.3. PPOA-N-Ac-2-Cl Inhibits Actin Ring Formation and Bone Resorption Activity

**Figure 4:**
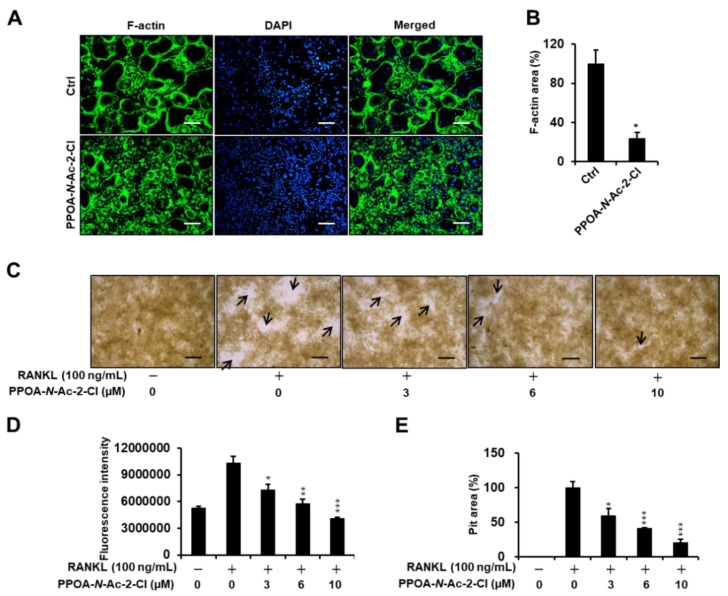
PPOA-*N*-Ac-2-Cl suppresses F-actin ring formation and bone resorption activity of osteoclasts. (**A**) Visualization of actin-ring formation in M-CSF and RANKL-induced osteoclasts in the presence of 6 µM PPOA-*N*-Ac-2-Cl or control. Scale bar = 200 μm. (**B**) The area of the actin belt was measured using ImageJ. The data shown are representative of at least three independent experiments. (**C**–**E**) BMMs were seeded on a fluoresceinamine-labeled calcium phosphate plate and treated with various concentrations of PPOA-*N*-Ac-2-Cl for 6 days. Scale bar = 200 μm. Black arrow indicates resorbed area by osteoclasts. After 6 days, the fluorescence intensity in each group was measured, at excitation wavelength of 485 nm and emission wavelength of 535 nm, using a fluorometric plate reader (**D**). Resorption pit areas were measured using Image J (**E**). * *p* < 0.05, ** *p* < 0.01, and *** *p* < 0.001 versus vehicle-treated control, 0 μM.

Previous reports have suggested that actin-ring formation is a visual phenotype of mature osteoclasts in bone resorption [17]. Hence, we conducted immunofluorescence analysis to further investigate the effect of PPOA-*N*-Ac-2-Cl on the formation of the actin ring (Figure 4A) in vitro. Most of the osteoclasts exhibited well-formed actin rings upon stimulation with RANKL and M-CSF, whereas the size and number of F-actin rings decreased significantly when the cells were treated with RANKL in the presence of 6 μM PPOA-*N*-Ac-2-Cl (Figure 4A). Next, we explored the effect of PPOA-N-Ac-2-Cl on osteoclast bone resorption in vitro using a bone resorption assay kit and visualized the resorbed area using light microscopy (Figure 4C). The results showed that, compared to the negative control consisting of only M-CSF treatment, M-CSF and RANKL stimulation markedly increased the number and size of bone resorption area (arrows, Figure 4C). The percentage of bone resorption area showed significant decrease upon treatment with 3, 6, and 10 µM PPOA-*N*-Ac-2-Cl. These results indicated that PPOA-*N*-Ac-2-Cl-mediated inhibition of bone resorption was attributed to the impairment of actin-ring formation. 

### 2.4. PPOA-N-Ac-2-Cl Downregulates Osteoclast Marker Genes Expression during Osteoclastogenesis 

**Figure 5:**
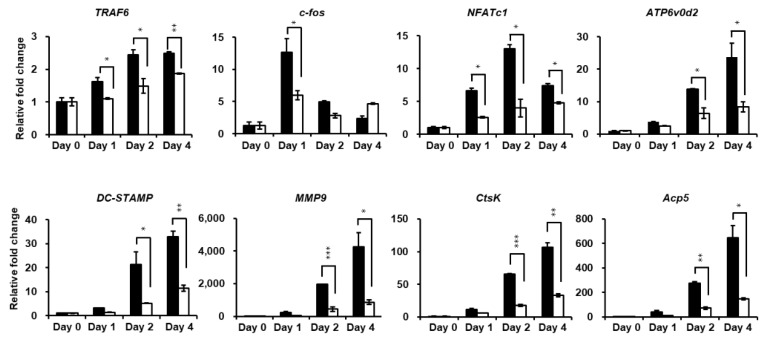
PPOA-*N*-Ac-2-Cl inhibits RANKL-induced osteoclast-specific gene expression in vitro. Relative mRNA expression of osteoclast-specific genes, *TRAF6*, *c-fos*, *NFATc1*, *ATP6v0d2*, *DC-STAMP*, *MMP9*, *CtsK*, and *Acp5 (TRAP)*, was analyzed by qRT-PCR in the presence or absence of treatment with 6 µM PPOA-*N*-Ac-2-Cl for 24 h (day 1), 48 h (day 2), or 96 h (day 4). Black bars indicate control, without PPOA-*N*-Ac-2-Cl treatment, and white bars indicate PPOA-*N*-Ac-2-Cl-treated group. Transcript levels were normalized to the expression levels of the control at day 0. The primers used for this experiment are listed in Table S1. * *p* < 0.05, ** *p* < 0.01, and *** *p* < 0.001 indicate the statistically significant difference between control and 6 µM PPOA-*N*-Ac-2-Cl treatment on each day.

Quantitative real-time PCR (qRT-PCR) was performed to further investigate the attenuation of osteoclastogenesis by PPOA-*N*-Ac-2-Cl. Figure 5 shows that the expression of the mRNA-encoding osteoclast-specific marker genes, such as *TRAF6*, *NFATc1*, *ATP6v0d2*, *DC-STAMP*, *Acp5*, and *CtsK*, increased markedly upon induction of osteoclast differentiation, showed in the control group. PPOA-*N*-Ac-2-Cl significantly suppressed RANKL-induced transcription of these genes, further supporting the inhibitory effect of PPOA-*N*-Ac-2-Cl on RANKL-induced osteoclast formation and function, as mentioned above.

### 2.5. PPOA-N-Ac-2-Cl Suppresses the Phosphorylation of MAPK and NF-κB 

The observations from Figure 3 suggested that PPOA-*N*-Ac-2-Cl suppressed RANKL-induced osteoclastogenesis at the early stages. A previous study reported that RANKL-induced MAPK/AP-1 activation and RANKL-mediated NF-κB signaling pathways are among the very early molecular events of osteoclast differentiation [18].

**Figure 6:**
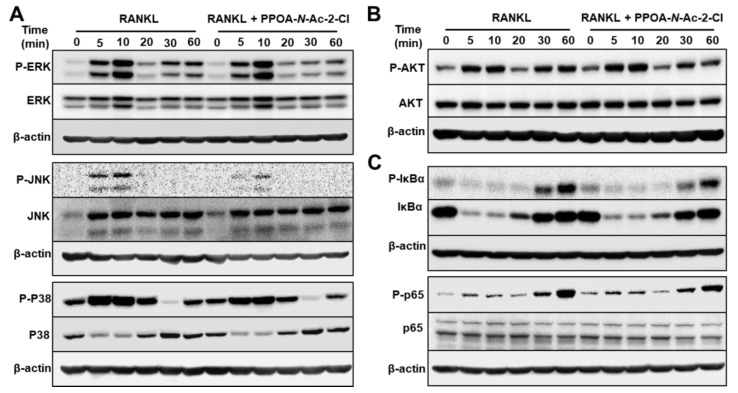
PPOA-*N*-Ac-2-Cl attenuates osteoclastogenesis via inhibition of MAPK and IκBa/p65 (NF-κB) signaling pathways. (**A**–**C**) BMMs were stimulated with 100 ng/mL RANKL for 0, 5, 10, 20, 30, or 60 min after pretreatment with 6 μM PPOA-*N*-Ac-2-Cl or DMSO for 2 h, and the phosphorylation of MAPKs, Akt, IκBa, and p65 was analyzed by immunoblotting. The densitometry graphs for the blots are shown in Figure S3. β-actin was used as the loading control.

To identify the effect of PPOA-*N*-Ac-2-Cl on the possible signaling cascades related to osteoclast formation and to confirm that PPOA-*N*-Ac-2-Cl inhibited osteoclast formation at the early stages of differentiation, phosphorylation of MAPKs and NF-κB was analyzed (Figure 6). BMMs were pre-treated with 6 μM of PPOA-*N*-Ac-2-Cl for 2 h and stimulated with RANKL for the indicated durations of time. As shown in Figure 6, PPOA-*N*-Ac-2-Cl reduced the phosphorylation of ERK, JNK, and p38 relative to total ERK, JNK, and p38 in BMMs after RANKL treatment. Degradation of I-κB, a component of NF-κB pathway, was not altered, whereas both phospho-I-κB and phospho-p65 showed significant reduction at 30 or 60 min after PPOA-*N*-Ac-2-Cl treatment. Collectively, these results suggested that PPOA-*N*-Ac-2-Cl represses both MAPK and NF-κB signaling in RANKL-induced osteoclast differentiation.

### 2.6. PPOA-N-Ac-2-Cl Suppresses the Expression of TRAF6

**Figure 7:**
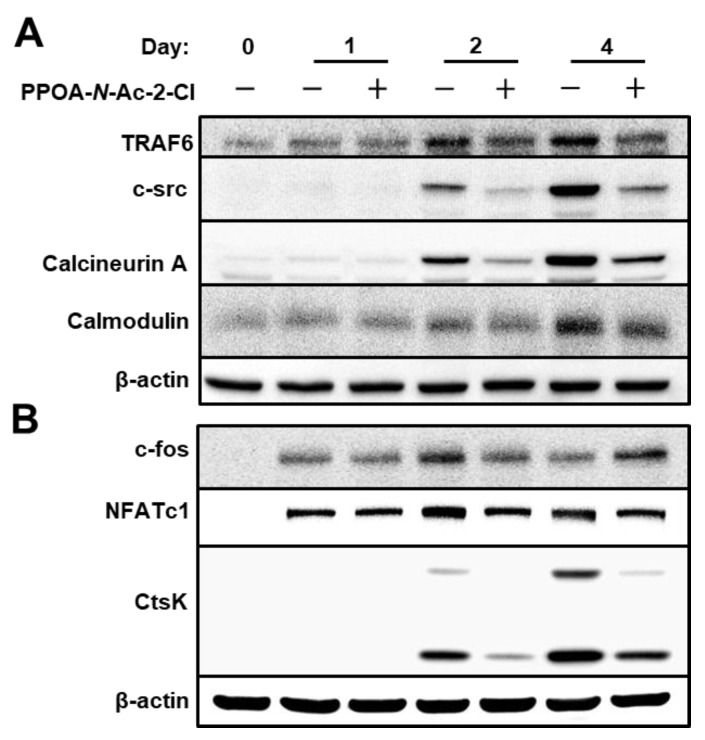
PPOA-*N*-Ac-2-Cl suppresses the expression of TRAF6. (**A**,**B**) BMMs were cultured with 6 μM PPOA-*N*-Ac-2-Cl for 0, 1, 2, or 4 days in the induction medium. Cell lysates were prepared and immunoblotting was performed with the indicated antibodies. The densitometry graphs for the blots are shown in Figure S4.

To further explore the mechanism by which PPOA-*N*-Ac-2-Cl suppresses osteoclastogenesis and bone resorption, we examined the expression of osteoclast-specific proteins (Figure 7). NFATc1 is known to be the master transcriptional regulator of osteoclast differentiation [19]. TRAF6 is known to be a pivotal component of the RANKL-RANK signaling pathway and an activator of the NF-κB and MAPK signaling [20]. As depicted in Figure 7A, TRAF6 expression was significantly upregulated during RANKL-induced osteoclast differentiation. However, upon treatment with PPOA-*N*-Ac-2-Cl, TRAF6 protein expression decreased significantly; the same trend was observed for the mRNA expression of TRAF6, as shown in Figure 5. Moreover, the protein levels of CtsK, a marker for bone resorption activity, were also suppressed upon treatment with PPOA-*N*-Ac-2-Cl, which was consistent with the results obtained from qRT-PCR analysis, as shown in Figure 5. In addition, the expression of proteins associated with osteoclast differentiation, downstream to TRAF6, was also suppressed upon PPOA-*N*-Ac-2-Cl treatment. Interestingly, c-fos expression levels were higher in PPOA-*N*-Ac-2-Cl-treated group compared to those in the control at day 4. In the PPOA-*N*-Ac-2-Cl treatment group, the expression of c-fos was continuously elevated from day 1 to day 4. These results suggested that PPOA-*N*-Ac-2-Cl-treated cells may start to differentiate into osteoclasts at day 4, while control cells initiate at day 2. In conclusion, our findings illustrate that PPOA-*N*-Ac-2-Cl suppresses osteoclast formation and bone resorption activity by the downregulation of TRAF6.

## 3. Discussion

Osteoclast-induced bone resorption and osteoblast-mediated bone formation are vital events that contribute to bone homeostasis [21]. Altered osteoclast formation can lead to excessive bone resorption, which can induce a number of bone-related diseases, such as osteoporosis and rheumatoid arthritis [18,22]. Therefore, osteoclasts have been regarded as the main target of anti-resorptive drugs [21]. Molecules containing *N*-(piperazine-1-yl)phenyl-2-phenoxyacetamide (PPOA) have been suggested to exhibit several biological and pharmacological properties, such as antitumor and tyrosine-sulfate mimetic activities [13,14,15]. However, the inhibitory effects of this series of compounds on osteoclastogenesis and their molecular mechanism(s) are still unknown. In this study, we demonstrate for the first time, to our knowledge, that PPOA-*N*-Ac-2-Cl inhibits osteoclastogenesis and bone resorption by downregulation of TRAF6 in vitro.

TRAF6 regulates innate and adaptive immune responses. TRAF6 protein is found to express in most tissues, including the heart, central nervous system, and muscle [23,24,25,26]. TRAF6 null mice develop severe osteopetrosis, spleen enlargement, hepatomegaly, and cardiomegaly [23,24,25]. TRAF6 expression and its ubiquitination are upregulated in cancer cachexia, cardiac ischemia/reperfusion injury and myocardial infarction. Therefore, regulation of TRAF6 expression by treatment with PPOA-*N*-2-Cl may be valuable not only for restoring bone homeostasis, but also in developing therapeutic strategies for other medical conditions.

To evaluate the function of PPOA derivatives on osteoclastogenesis, a biological screening of PPOA derivatives was performed. We found that PPOA-*N*-Ac-2-Cl was able to inhibit osteoclast formation, in a dose-dependent manner, in the presence of M-CSF and RANKL. In addition, treatment with PPOA-*N*-Ac-2-Cl for different periods during osteoclast differentiation led to a significant inhibition in the size of osteoclasts upon TRAP staining (Figure 3), suggesting that PPOA-*N*-Ac-2-Cl attenuated osteoclastogenesis in the early stages of osteoclast differentiation. Furthermore, as shown in Figure 4, compared to ctrl, PPOA-*N*-Ac-2-Cl also caused marked suppression of F-actin ring formation and bone resorption activity, indicating that it affects both osteoclast formation and bone resorption. However, our results are not sufficient to prove the direct inhibition of bone resorption activity by treatment with PPOA-*N*-Ac-2-Cl. We speculate that PPOA-*N*-Ac-2 Cl inhibits developing mature osteoclasts at the early stage, and the reduced number of mature osteoclasts leads to low bone resorption activity under PPOA-*N*-Ac-2-Cl treatment.

TRAF6 is an initiator of osteoclastogenesis, and further activates MAPKs and NF-κB signaling and/or recruits c-Src [27,28,29]. Previous studies have shown that c-Src recruitment can activate Ca^2+^ signaling [18]. The oscillations in intracellular Ca^2+^ levels can stimulate the activation of calmodulin and calcineurin, which subsequently results in the self-amplification and nuclear translocation of NFATc1 [28,29]. Our results showed that treatment with PPOA-*N*-Ac-2-Cl significantly inhibited the expression of *TRAF6* and the protein levels of c-Src and calcineurin (Figure 7), suggesting that PPOA-*N*-Ac-2-Cl might impact osteoclastogenesis and bone resorption by suppressing the activation of the TRAF6/c-Src/Ca^2+^ pathway. NF-κB signaling plays a vital role in RANKL-induced osteoclast differentiation [21,30,31]. In the classical NF-κB signaling pathway, IKK complex is activated, leading to the phosphorylation of IκBα, which is degraded via the ubiquitin proteasome system [32]. In the IκB-independent signaling pathway, NF-κB is directly phosphorylated by IKK, which modulates the transcription of NF-κB [33]. We observed that treatment with PPOA-*N*-Ac-2-Cl showed an inhibitory effect on the phosphorylation of the NF-kB subunit p65, but had no effect on the expression of IκBα. These results indicate that the suppression of osteoclastogenesis by PPOA-*N*-Ac-2-Cl might be due to the suppression of NF-kB activation, but this occurs without altering the degradation of IκB. 

The activation of MAPKs can synergistically cause the activation of NFATc1 [34], which is a master transcription factor for osteoclast differentiation regulating several osteoclast-specific genes, such as *Acp5*, *DC-STAMP, OC-STAMP*, *ATP6v0d2*, *CtsK*, and *MMP9*, playing an essential and unique role in the fusion of osteoclast precursors and bone resorption [35,36,37]. Our analysis of MAPKs signaling showed that the expression levels of p-ERK1/2, p-JNK, and p-p38 were decreased upon PPOA-*N*-Ac-2-Cl treatment. Previous evidence showed that RANKL-induced MAPKs/activator protein-1(AP-1) activation occurs during the very early stage of osteoclastogenesis [18,22]. Therefore, we determined the stages of osteoclastogenesis that were affected by treatment with PPOA-*N*-Ac-2-Cl. As expected, PPOA-*N*-Ac-2-Cl repressed RANKL-induced osteoclast differentiation only at the early stages (Figure 3). JNK phosphorylates the transcription factor c-Jun to form the AP-1 complex with c-fos, which is an important transcription factor in osteoclastogenesis [18]. The suppressed levels of c-fos by PPOA-*N*-Ac-2-Cl on day 2 may have led to the downregulation of NFATc1 expression at day 2 and day 4 (Figure 7), suggesting that AP-1 expression levels are important for NFATc1-mediated signaling cascade.

In spite of the novel findings in this study, it still merits further exploration and discussion. Bone homeostasis is a complex phenomenon and targeting both bone resorption and osteoblastic bone formation is essential in the treatment of bone diseases [27,38]. However, in this study, we mainly focused on exploring the mechanism(s) of the inhibitory effects of PPOA-*N*-Ac-2-Cl on osteoclast formation and osteoclast-related bone resorption. Our results show that PPOA-*N*-Ac-2-Cl might attenuate the osteoclastogenesis and bone resorption via the downregulation of TRAF6; further studies are needed to address the effect of PPOA-*N*-Ac-2-Cl on osteoblasts and its possible mechanism of action. 

Collectively our findings revealed that PPOA-*N*-Ac-2-Cl attenuated RANKL-induced osteoclastogenesis via down-regulation of TRAF6, and reduction in MAPKs and p65 activation and c-Src expression. Repression of the MAPK, NF-κB, and c-Src signaling pathways led to the downregulation of NFATc1, which resulted in inhibition of the expression of osteoclast-related marker genes, including *Acp5*, *DC-STAMP*, *ATP6v0d2*, *CtsK*, and *MMP9*. Hence, we conclude that PPOA-*N*-Ac-2-Cl has the potential to be a small molecule anti-resorptive drug candidate for osteoclast-related disorders, although its in vivo effect on bone loss needs to be further investigated.

## 4. Materials and Methods 

### 4.1. Reagents and Antibodies 

PPOA-*N*-*Ac*-2-Cl, purchased from ChemBridge (San Diego, CA, USA), was dissolved in dimethyl sulfoxide (DMSO; Sigma-Aldrich, St. Louis, MO, USA) to obtain a 50-mM solution. Aliquots of the solution were stored at −20 °C and diluted to the appropriate concentrations in cell culture medium immediately before use. DMSO was used as the vehicle control. Alpha-modified minimal essential medium (α-MEM) and fetal bovine serum (FBS) were purchased from Thermo Fisher Scientific (Waltham, MA, USA). Bone resorption assay kit was procured from Cosmo Bio Co., Ltd., Tokyo, Japan. BCA protein assay kit was purchased from Pierce Biotechnology, (Rockford, IL, USA). Primary antibodies including anti-β-actin (Sigma-Aldrich, St Louis, MO, USA), anti-c-Src (Santa Cruz Biotechnology, Inc., Dallas, TX, USA), anti-cathepsin K (Santa Cruz Biotechnology, Inc., Dallas, TX, USA), anti-ERK1/2, anti-phospho-ERK1/2, anti-AKT, anti-phospho-AKT, anti-IκBa, anti-phospho-IκBa, anti-p38, anti-phospho-p38, anti-JNK, anti-phospho-JNK, anti-c-fos, anti-NFATc1, anti-p65, and anti-phospho-p65, anti-TRAF6, anti-calcineurinA, anti-calmodulin were purchased from Cell Signaling Technology (Boston, MA, USA). Horseradish peroxidase (HRP)-conjugated secondary antibodies were obtained from Cell Signaling Technology, and chemiluminescence signals were detected using an ECL system (iNtRON, Seoul, Korea). 

### 4.2. Bone Marrow-Derived Macrophage Isolation and Culture 

For osteoclast differentiation in vitro, bone marrow-derived macrophages (BMMs) were prepared as previously described [39]. Briefly, fresh mouse bone marrow cells were isolated from the femur and tibiae of 8-week-old C57BL/6J mice, by flushing the bone marrow with α-MEM. Flushed cells were incubated in culture medium (α-MEM containing 10% heat-inactivated fetal bovine serum (FBS) and 1% penicillin/streptomycin) for 1 day. The non-adherent cells were then collected and cultured in Petri dishes stimulated with 30 ng/mL M-CSF (PeproTech, Rocky Hill, NJ, USA) to obtain BMMs. Three days later, culture supernatants were discarded, and the adherent cells were further cultured in induction medium, supplemented with 30 ng/mL M-CSF and 50 ng/mL RANKL (PeproTech). Culture medium was replaced every 2 days until the osteoclasts formed. Next, the differentiated BMMs were fixed with 3.7% formaldehyde in phosphate-buffered saline (PBS) for 20 min and stained for TRAP using a TRAP-staining kit (Sigma-Aldrich). The osteoclasts were counted based on the number of nuclei (*n* ≥ 3) under a microscope. Each osteoclast formation assay was performed independently at least three times. All the mice were housed in a specific pathogen-free facility and all animal experiments were approved by IACUC at Chonnam National University (Approval number CNU IACUC-YB-2017-70, on 31 October 2017).

### 4.3. Cell Viability Assay

Cytotoxicity of PPOA-*N*-Ac-2-Cl was evaluated using MTT assay, as previously described [40,41]. BMMs were cultured in black 96-well polypropylene plates (Thermo Scientific Nunc, Waltham, MA, USA) in the presence of 30 ng/ml M-CSF, with or without PPOA-*N*-Ac-2-Cl for 3 days. After 3 days, MTT (0.5 mg/mL final concentration) was added to each well, and the cells were incubated for 4 h, followed by addition of 100 μL 10% DMSO, followed by incubation for 3 h. The optical density (OD) was measured using a SpectraMax i3x microplate reader at 450 nm (Molecular Devices, San Jose, CA, USA). 

### 4.4. F-actin Ring Formation Assay

F-actin ring in osteoclasts was detected by staining with rhodamine-conjugated phalloidin (Thermo Fisher Scientific, MA, USA). The BMMs were cultured in 12-mm cover glasses in the presence of M-CSF (30 ng/mL) and RANKL (50 ng/mL), with or without PPOA-*N*-Ac-2-Cl (6 μM). After the formation of osteoclasts, the cells were fixed for 15 min using 3.7% paraformaldehyde and blocked for 45 min using 5% bovine serum albumin. The osteoclasts were then stained with rhodamine-conjugated phalloidin to observe the F-actin rings. The cells were then washed with PBS, stained with DAPI, and visualized using a fluorescence microscope.

### 4.5. Resorption Pit Assay

Bone resorption assay was performed using a bone resorption assay kit (Cosmo Bio Co., Ltd., Tokyo, Japan), according to the manufacturer’s directions. BMMs were seeded in a bone resorption assay plate (2 × 10^4^ cells/well) in the presence of M-CSF (30 ng/mL). The calcium phosphate-coated plate contains fluoresceinamine-labeled chondroitin sulfate, which was released as the osteoclasts degraded the calcium phosphate on the plate. Twenty four hours later, cells were stimulated with M-CSF (30 ng/mL) and RANKL (100 ng/mL) and treated with or without the indicated concentrations of PPOA-*N*-Ac-2-Cl, until mature osteoclasts formed (about 5 days). On the following day, the culture supernatant was harvested into a 96-well black polypropylene micro-well plate (Thermo Scientific Nunc, Waltham, MA, USA) and mixed with 50 μL 0.1 N NaOH. The fluorescence intensity was then measured using the SpectraMax i3x fluorescence plate reader at the excitation and emission wavelengths of 485 nm and 535 nm, respectively. In addition, the resorption areas were calculated by analyzing 10 randomly selected pictures per well (taken at 10× magnification) using ImageJ software (version 1.41, https://imagej.nih.gov/ij), as previously described [39].

### 4.6. RNA Isolation and Quantitative Real-Time PCR

BMMs were plated in 6-well plates, at the density of 3 × 10^5^ cells/well, in the presence of M-CSF and RANKL, with or without PPOA-*N*-Ac-2-Cl for 4 days. As described in our previous study [39,42], QIAzol RNA Lysis reagent (Qiagen Sciences, Valencia, CA, USA) was used to isolate the total RNA from the BMMs. The cDNAs were then synthesized using a PrimeScript™ RT Reagent Kit for qRT-PCR (Takara Biotechnology, Tokyo, Japan), following the manufacturer’s instructions. qRT-PCR was performed using a QuantStudio 3 real-time PCR system (Applied Biosystems, Foster City, CA, USA) with a Power SYBR Green PCR Master Mix (Applied Biosystems, Foster City, CA, USA) and the standard temperature protocol. The results obtained using a cycle threshold were expressed as relative ratios and calculated using the 2^−ΔΔ*C*T^ method (expressed as the relative fold ratio). The expression levels were normalized to glyceraldehyde 3-phosphate dehydrogenase (GAPDH) expression. Three separate experiments were performed. Table S1 lists the primers used for real-time PCR assay.

### 4.7. Western Blot Assays

To detect the expression of osteoclastogenesis-related proteins and explore the possible signaling pathways involved, osteoclast cells were lysed in chilled lysis buffer [50 mM Tris–HCl (pH 7.5), 150 mM NaCl, 1% NP-40, 0.5% sodium deoxycholate, 0.1% sodium dodecyl sulfate (SDS), and 2 mM EDTA, and protease inhibitors]. The supernatants were collected following centrifugation (16,400× *g*, 4 °C, 30 min). After determining the concentrations of protein extracted from the differentiated osteoclasts using BCA protein assay (Pierce Biotechnology), protein samples were separated using 12% polyacrylamide gel electrophoresis and then transferred to a polyvinylidene fluoride membrane. The membrane was blocked in 5% skim milk or 3% BSA for 1 h, followed by incubation with primary antibodies (1:1000) at 4 °C overnight. After washing three times with TBST, the membrane was incubated with HRP-conjugated secondary antibodies (1:2000) for 1 h and the chemiluminescence signals were detected using an ECL reagent (iNtRON, Seoul, Korea), according to the manufacturer’s instructions.

### 4.8. Statistical Analysis

Statistical analyses were performed using unpaired two-tailed Student’s *t*-tests (* *p* < 0.05; ** *p* < 0.01; *** *p* < 0.001; NS, not significant). All data are expressed as the mean ± standard deviation (SD). Results are representative examples of more than three independent experiments.

## Supplementary Materials

Supplementary materials can be found at https://www.mdpi.com/1422-0067/20/20/5196/s1. Figure S1. Structures of PPOA derivatives, (A) acetamide A, (B) acetamide B, (C) acetamide C, and (D) acetamide D. Figure S2. Cell viability assay. MTT assay was performed to determine the cell viability using the indicated concentrations of PPOA-*N*-Ac-2-Cl for the indicated durations of time with M-CSF (30 ng/mL). Figure S3. Quantification of the ratios of band intensity of p-ERK, p-JNK, p-P38, p-AKT, p-P65, and p-IκBα relative to their total forms that total ERK, JNK, P38, AKT, P65, and IκBα. Black bars indicate control, without PPOA-*N*-Ac-2-Cl treatment, and white bars indicate PPOA-*N*-Ac-2-Cl-treated group. * *p* < 0.05 versus the vehicle-treated control group. Figure S4. Quantification of the ratios of band intensity of TRAF6, c-src, Calcineurin A, Calmodulin, c-fos, NFATc1, and CtsK relative to β-actin. Black bars indicate control, without PPOA-*N*-Ac-2-Cl treatment, and white bars indicate PPOA-*N*-Ac-2-Cl-treated group. * *p* < 0.05 versus the vehicle-treated control group. Table S1. Primers used in the study.
