# Supplementary material for: Inhibitory Effects of N-[2-(4-acetyl-1-piperazinyl) phenyl]-2-(2-chlorophenoxy) acetamide on Osteoclast Differentiation In Vitro via the Downregulation of TRAF6
Source: Int J Mol Sci. 2019 Oct 20;20(20):5196. doi: 10.3390/ijms20205196 (PMC6829416; doi:10.3390/ijms20205196)
Supplement: Supplementary file 1 [file ijms-20-05196-s001.pdf]

Supplementary

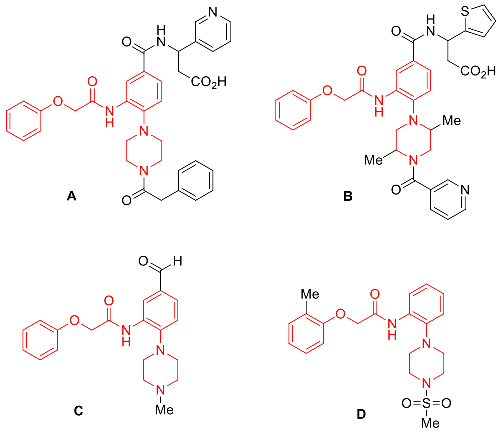

Figure S1. Structures of PPOA derivatives, (**A**) acetamide ***A***, (**B**) acetamide ***B***, (**C**) acetamide ***C***, and (**D**) acetamide ***D***.

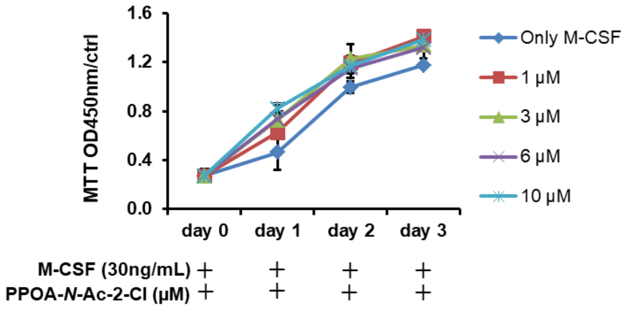

Figure S2. Cell viability assay. MTT assay was performed to determine cell viability using the indicated concentrations of PPOA-*N*-Ac-2-Cl for the indicated durations of time with M-CSF (30 ng/mL).

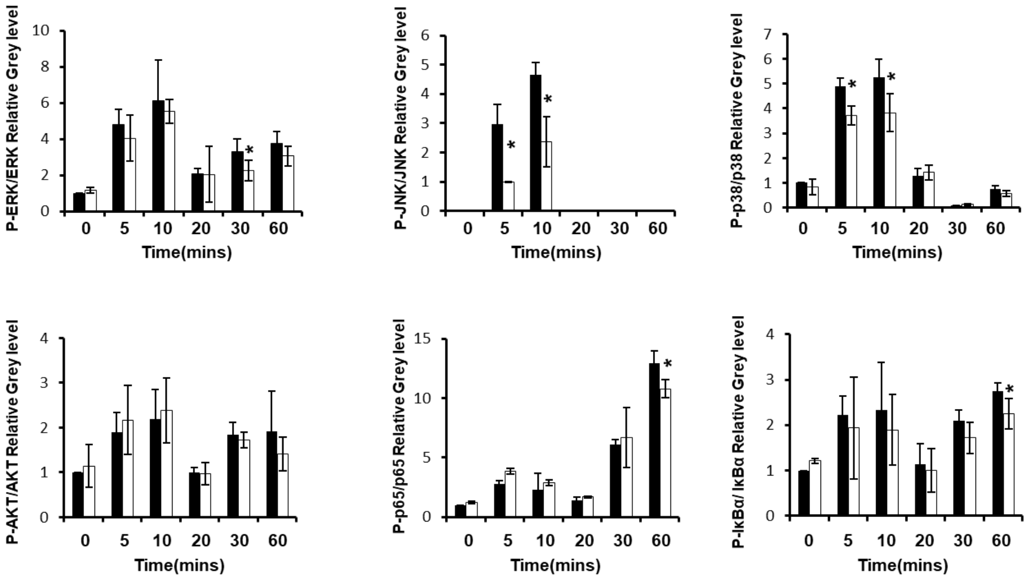

Figure S3. Quantification of the ratios of band intensity of p-ERK, p-JNK, p-P38, p-AKT, p-P65 and p-IκBα relative to their total forms that total ERK, JNK, P38, AKT, P65 and IκBα. Black bars indicate control, without PPOA-*N*-Ac-2-Cl treatment, and white bars indicate PPOA-*N*-Ac-2-Cl-treated group. * $p < 0.05$ versus the vehicle-treated control group.

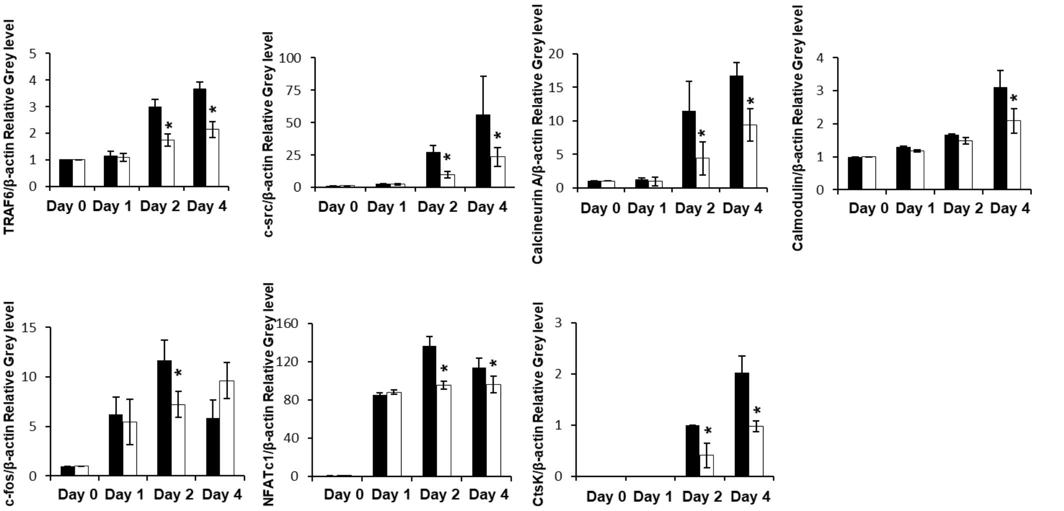

Figure S4. Quantification of the ratios of band intensity of TRAF6, c-src, Calcineurin A, Calmodulin, c-fos, NFATc1 and CtsK relative to β-actin. Black bars indicate control, without PPOA-*N*-Ac-2-Cl treatment, and white bars indicate PPOA-*N*-Ac-2-Cl-treated group. * $p < 0.05$ versus the vehicle-treated control group.

Table S1. Primers used in the study.

Gene		Primer sequence (5′→3′)
Cathepsin K	Forward	GGACGCAGCGATGCTAACTAA
	Reverse	CAGAGAGAAGGGAAGTAGAGTTGTCACT
Acp5	Forward	CAGCTGTCCTGGCTCAAAA
	Reverse	ACATAGCCCACACCGTTCTC
DC-STAMP	Forward	CGCACGATGCTTCATTCTTC
	Reverse	CAGTGCCAGCCGCAATC
GAPDH	Forward	TGTGTCCGTCGTGGATCTGA
	Reverse	GATGCCTGCTTCACCACCTT
MMP9	Forward	CTGGACAGCCAGACACTAAAG
	Reverse	CTCGCGGCAAGTCTTCAGAG
NFATc1	Forward	ACCACCTTTCCGCAACCA
	Reverse	GGTACTGGCTTCTCTTCCGTTTC
ATP6v0d2	Forward	GTGAGACCTTGGAAGACCTGAAA
	Reverse	TCCTCATCTCCGTGTCAATTTTG
c-fos	Forward	CGAAGGGAACGGAATAAGATG
	Reverse	GCTGCCAAAATAAACTCCAG
TRAF6	Forward	TCGGACCCTGGAGGACAA
	Reverse	CCAAACTTGCCAATCTTCCAA
